# Adsorption Behavior and Mechanism for the Uptake of Fluoride Ions by Reed Residues

**DOI:** 10.3390/ijerph15010101

**Published:** 2018-01-09

**Authors:** Rong Song, Shengke Yang, Haiyang Xu, Zongzhou Wang, Yangyang Chen, Yanhua Wang

**Affiliations:** 1 Key Laboratory of Subsurface Hydrology and Ecological Effects in Arid Region, Ministry of Education, Chang’an University, Xi’an 710054, China; songrong0811@126.com (R.S.); ysk110@126.com (S.Y.); zongzhouwang@126.com (Z.W.); 18291960210@163.com (Y.C.); 2 School of Environmental Science and Engineering, Chang’an University, Xi’an 710054, China; 3 Liaoning Zhongwang Group Co., Ltd., Liaoyang 111003, China; 13386793382@163.com; 4 School of Geography and Tourism, Shaanxi Normal University, Xi’an 710054, China

**Keywords:** fluoride, reed, adsorption, desugared

## Abstract

The adsorption behavior and mechanism for the uptake of fluoride ions by untreated and desugared reed residues (roots, stems and leaves) were studied through adsorption experiments, elemental analysis, infrared spectroscopy and surface area analysis. The results showed that the adsorption capacity of untreated and desugared reeds followed the order: desugared roots 2136 mg/kg > desugared leaves 1825 mg/kg > desugared stems 1551 mg/kg > untreated roots 191 mg/kg > untreated stems 175 mg/kg > untreated leaves 150 mg/kg, so adsorption capacity of desugared reeds was larger than that of the untreated reeds. The adsorption kinetic of fluoride ions followed a pseudo-first-order model. A Langmuir model could be used to fit the isothermal adsorption process which was a spontaneous endothermic reaction involving mainly physical adsorption. The ΔG for the uptake of fluoride by the desugared reeds was more negative, so the degree of spontaneity was higher than for the use of the untreated reeds. After samples were desugared, the specific surface area and aromaticity of the reed increased, while the polarity and hydrophilicity decreased, which explained the adsorption amount of desugared reed was higher than that of the untreated. This study enriches techniques and methods of removing fluoride ions from water.

## 1. Introduction

Fluorine is a non-metallic chemical element, widely distributed in the environment in the atmosphere [1], minerals [2,3,4,5], underground [6,7], food [8,9,10] and groundwater [11]. Excessive fluoride concentrations in drinking water and food can lead to endemic fluorosis. In recent years, many measures and processes for the purification of high fluoride water have been evaluated. However the lack of an economic and practical high-fluoride water purification technology has created a technical and economic bottleneck in implementing a strategy safe water for supplying safe drinking water to remote and poor areas. This has restricted the development of the social economy in those areas. The treatment techniques of high fluoride water are mainly reverse osmosis, ion-exchange, chemical precipitation, membrane filtration and adsorption [12]. Biological adsorption methods are widely studied due to the high efficiency of removal fluoride and environmental friendliness [13]. Therefore, investigating the adsorption behavior and mechanism of fluoride ion is of great necessity.

Natural zeolites, the macromolecule chitosan, soil and other natural materials are cheap and easy to obtain, but their adsorption capacity towards fluoride ions is quite low. The adsorption of fluoride ions by natural material is usually limited to materials modified with metal compounds. After modification, the adsorption capacity can be improved, but the modification may cause secondary pollution problems. Sun et al. [14] reported modified zeolite could reduce the concentration of fluoride in wastewater from 10 mg/L to 1.0 mg/L. Hong et al. [15], and Liang et al. [16] reported that the adsorption capacity of chitosan modified by lanthanum was up to 3.67 mg/g, and the chitosan modified by misch metal (an alloy of rare-earth metals) has higher adsorption capacity than chitosan modified by lanthanum. Wang et al. [17] showed that modification of clay with PAC improved its adsorption performance by 62.61%, while also enhancing the adaptability of clay to pH.

Plant residues are inexpensive, renewable and natural absorbent materials widely available in the natural environment. Plants are readily accessible in a lot of polluted areas, such as mining sites and river edges. Therefore, the study of the adsorption of pollutants by plants has gradually become a focal point in sorption science. Nowadays, domestic and foreign research on the adsorption of ions by plants has made some progress. Ma et al. [18] reported fluoride ions were mainly transported to leaves, accounting for 90% of the fluoride content of whole tea plants, while the roots and stems did not accumulate many fluoride ions. He et al. [19] showed that the distribution of fluoride content in spinach was the same as in celery, and in the order old leaf > young leaf > root. Liao studied the tolerance and absorption mechanism of hyperaccumulators towards heavy metals. Wang et al. [20] studied the adsorption characteristics of reed on the heavy metals Pb^2+^ and Mn^2+^, which showed different plant organs have different adsorption capacities for different ions. Studies on the adsorption of pollutants by reeds were mostly concentrated on organic pollutants and heavy metals and little on inorganic ions. 

Therefore, this study the uptake of fluoride ions by reed from the Weihe River was investigated in terms of its adsorption behavior and the mechanism, looking at different untreated and desugared reed tissues (roots, stems and leaves).

## 2. Materials and Methods

### 2.1. Sample Collection and Pre-Treatment

One kind of reed material was obtained from the Chanba Ecological District (Xi’an, China, 34°25′14.45″ N, 109°1′1.46″ E). The untreated root (R-U), stem (S-U), and leaf (L-U) samples were washed with deionized water to remove surface impurities such as dust, dried in the shade, broken into pieces and passed through a 60-mesh sieve, then stored in different clean sealed dehumidification jars.

**Figure 1:**
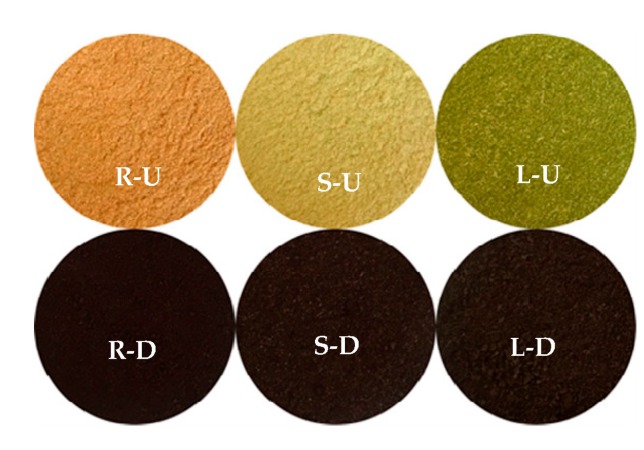
Untreated and desugared samples.

The desugarization process of the original samples was conducted by acidification with 6 mol/L HCl solution under reflux for 6 h below 100 °C [21], followed by extraction by vacuum filtration, and washing to neutrality with deionized water to obtain desugared root (R-D), stem (S-D), and leaf (L-D) samples (Figure 1), which finally were stored in the same way as the untreated samples above.

The initial fluoride ion solution was prepared by adding KF (Guaranteed Reagent, GR). An ion selective electrode method was used to determine the fluoride ion content.

### 2.2. Adsorption Experiment Methods

The initial concentration of fluoride ion for kinetic adsorption experiments was 2.4 mg/L at 298 K. In all experiments the same amount of plant samples was used. The concentration of fluoride ions remaining in the solution was determined at regular intervals, then adsorption curves were drawn and kinetic adsorption models were fitted. Isothermal adsorption experiments were conducted at 298 K with the concentration of fluoride ion solution was varied between 2.0 mg/L to 10.0 mg/L. The thermodynamics experiments were conducted at 298 K, 308 K and 318 K, pH = 7.0, the other steps followed isothermal adsorption experiments. Equations were fitted and calculated following methods described in the literature [22,23,24,25,26].

### 2.3. Analysis

Fourier transform infrared (FTIR) spectra of the untreated and desugared reed residues were recorded in the 4000–400 cm^−1^ region for a KBr pellet by a FTIR spectra (model Tensor-27, Bruker, Karlsruhe, Germany) with a resolution of 4.0 cm^−1^. Elemental (C, H, O, N) analyses of the untreated and desugared reed residues were conducted using a Vario EL cube elemental analyzer (Elementar, Frankfurt, Germany). The H/C, (N + O)/C and O/C molar ratio of reed residues were calculated to evaluate their aromaticity, polarity and hydrophilicity, respectively. The surface area, pore area, pore volume and average pore size determinations were carried out by N_2_ adsorption isotherms using a Micrometrics ASAP 2020K surface area analyzer (Micromeritics Instrument Corp., Atlanta, GA, USA) [27].

## 3. Results and Discussion

### 3.1. Characteristic of Adsorption Behavior 

#### 3.1.1. Characteristic of Kinetic Adsorption

**Figure 2:**
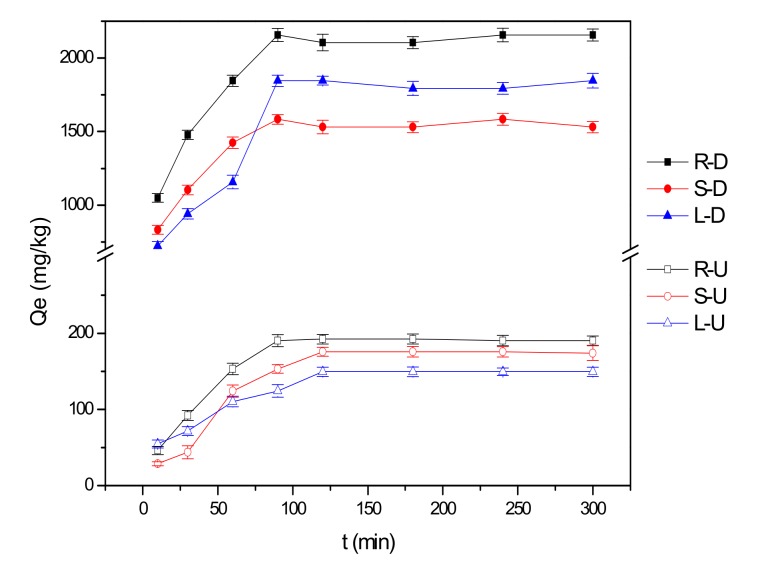
Adsorption kinetic curves for the uptake of fluoride by reed residues. R-U, S-U, L-U refer to the roots, stems, leaves of the untreated samples, and R-D, S-D, L-D refer to the roots, stems, leaves with desugarization.

The adsorption kinetic curves for the uptake of fluoride ions by reed residues are shown in Figure 2. All samples rapidly adsorbed the fluoride ions during the first 90 min of the experiment, and gradually reached saturation at about 120 min. This was due to the fact that the initial concentration of fluoride in the solution was higher and the adsorption was easier. With the passage of time, the adsorption sites of the adsorbent surface became occupied, so that the adsorption was blocked and the adsorption rate slowed down, gradually reaching equilibrium.

The fluoride absorption capacity of reed residues could be greatly improved by desugarization of the samples, which might be due to the fact this process increased the pore sizes and thus raised the surface area of the samples. In this study, the adsorption capacity of roots was highest, whether the samples were desugarized or not; the adsorption capacity of the untreated reed stems was higher than that of leaves, but the equilibrium adsorption capacity of the desugared reed leaves was higher than that of stems. The adsorption behavior of untreated and desugared reed towards fluoride ions was fitted according to the pseudo-first-order model and the pseudo-second-order model (Table 1). 

Listed are the equilibrium adsorption amount of roots, stems and leaves of untreated and desugared reeds (Table 1). The adsorption amount increased significantly by 8~12 times after desugarization, which showed that desugarization could promote the adsorption of fluoride ions by reed residues. Untreated samples were in good agreement with the pseudo-first-order model, but desugared samples were in good agreement with the pseudo-second-order model because correlation coefficient R^2^ of pseudo-second-order model was higher than that of pseudo-first-order model [28]. 

#### 3.1.2. Characteristic of Isothermal Adsorption

**Figure 3:**
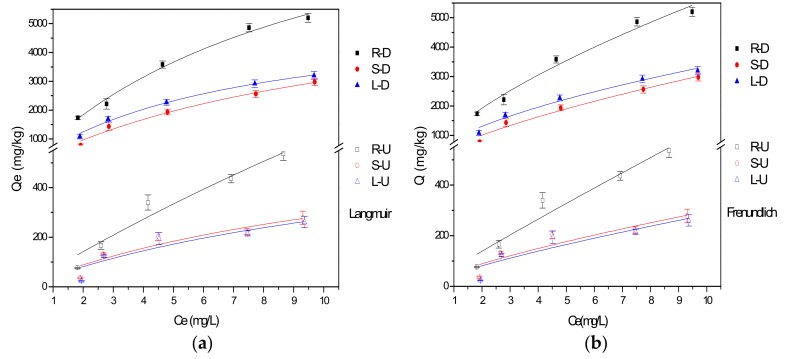
The fitted isothermal adsorption model for the uptake of fluoride ions by reed residues. (**a**) Langmuir Model; (**b**) Freundlich Model.

The data for the uptake of fluoride ions adsorbed by roots, stems and leaves of reed before and after desugarization were fitted according to Langmuir and Freundlich model, respectively. The fitted curves are shown in Figure 3, and the fitted parameters are shown in Table 2.

In isothermal adsorption process the adsorption capacity of fluoride ion before and after desugarization had significant differences (Figure 3), and the equilibrium adsorption capacity increased with rising equilibrium concentrations. The coefficient R^2^ of fitted Langmuir model of the fluoride ions adsorption by the untreated reed was 0.9401, 0.8418 and 0.8116, and the fitted Freundlich model R^2^ was 0.9351, 0.8181 and 0.7867. After desugarization, according to Langmuir model fitting, R^2^ was 0.9882, 0.9880 and 0.9917, and R^2^ of Freundlich model fitting was 0.9749, 0.9695 and 0.9737, respectively. Q_m_ and K_L_ of fitted Langmuir model increased significantly and K_F_ of Freundlich model fitting increased after desugarization. It can be seen that both the Langmuir model and the Freundlich model could describe the isothermal adsorption process of reed roots, desugared stems and leaves, but the Langmuir model provided a slightly better fit to the data. Comparative investigation of fluoride adsorption using different adsorbents is summarized in Table 3. Fluoride adsorption capacity of untreated roots in this study was similar to the natural materials in other literature while adsorption capacity of untreated stems and leaves was lower. After desugarization, the fluoride adsorption capacity of roots, stems and leaves increased significantly and reached levels comparable to modified and synthetic materials.

#### 3.1.3. Characteristic of Thermodynamic Adsorption

**Figure 4:**
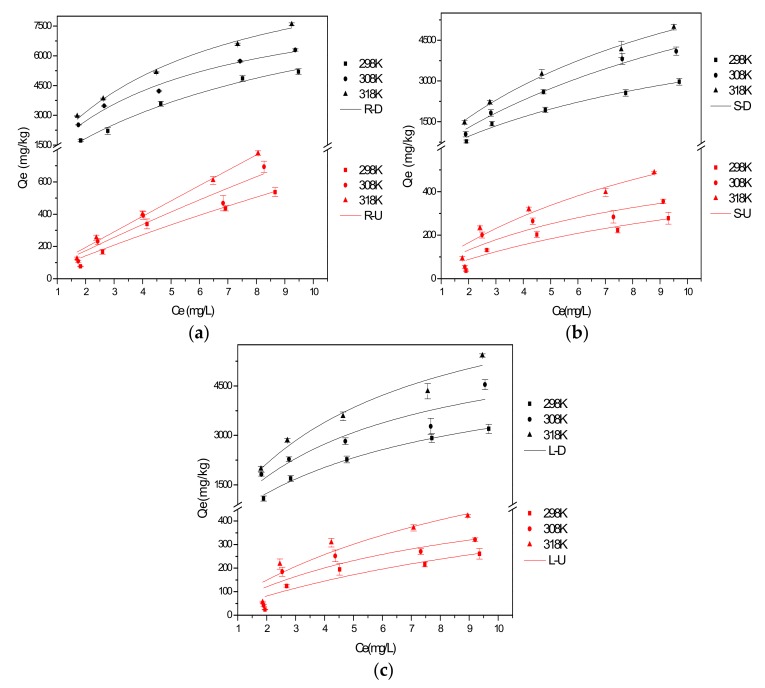
The fitted Langmuir model of the untreated and desugared (**a**) roots; (**b**) stems; (**c**) leaves at different temperatures.

Isothermal adsorption experiments were carried out at 298 K, 308 K and 318 K. The data was fitted to the Langmuir model. The fitted curves are shown in Figure 4 and the fitted parameters are listed in Table 4.

It can be seen from Figure 4 that with increasing temperature, the equilibrium adsorption capacity increased significantly. This suggested that the adsorption process was endothermic.

The thermodynamic parameters are shown in Table 5. K_0_ is an adsorption constant that is calculated by the intercept of graph of ln (Qe/Ce) versus Ce.

It can be seen from Table 5 that ΔG is less than 0 and greater than −20, indicating that the adsorption process was mainly a physical adsorption and spontaneous reaction. ΔG gradually decreased with increasing temperature, indicating that the increase of temperature was conducive to the spontaneous adsorption; ΔH > 0 indicated that the process of adsorption of fluoride ions was endothermic; ΔS > 0 indicated that in the adsorption process, the structure of reed surface was changed, so that the solid-liquid interface increased the degree of disorder. 

### 3.2. Analysis of Adsorption Mechanism

#### 3.2.1. Analysis of the Elemental Composition

The elemental composition and molar ratio of the reed samples are given in Table 6.

It can be seen from Table 5 that the reed residues contained large amounts of the elements C, H, and O. In desugared samples, carbon content was increased, while hydrogen and oxygen contents were decreased compared to the untreated reed residues. The aromatic, polar and hydrophilic index of roots decreased by 0.44, 0.24 and 0.23 units, respectively, after desugarization, and the aromatic index of the desugared reed stems and leaves all decreased by 0.24 units, polar index decreased by 0.12, 0.16 units, hydrophilic index decreased by 0.11, 0.13 units. This may be due to the removal of the sugar from the sample surface after desugarization and thus to removal of polar tissue. The most significant changes occurred in reed roots.

The adsorption coefficient K_d_ and the partition coefficient K_OC_ [41] of the organic carbon changed significantly during desugarization, and increasing compared with the untreated samples. The increase for the roots was the largest. The increases of the reed stems and leaves was similar. The increase of K_d_ and K_OC_ values corresponded to the increase in fluoride adsorption capacity of reed residues before and after desugarization.

#### 3.2.3. Analysis of FTIR 

**Figure 5:**
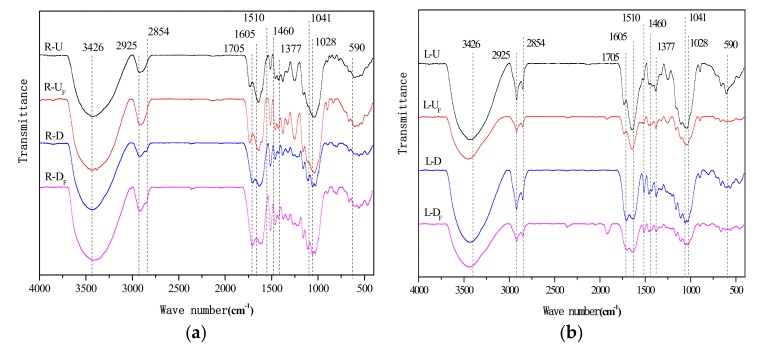
FTIR spectra of the reed (**a**) roots and (**b**) leaves.

FTIR spectra of the samples in the range of 4000–400 cm^−1^ before and after desugarization are presented in Figure 5.

The C-O-C band at 1300–1000 cm^−1^ indicates one of the main functional groups associated with sugars and carbohydrates as found in the untreated samples. Desugarization led to a significant drop in the band intensity in the 1300–1000 cm^−1^ wavenumber range of. Stretching vibrations of C=C, C=O and –COOH between 1750–1640 cm^−1^ were also affected, while the C-H stretching vibration (3000–2850 cm^−1^) and C-H bending vibration (1465–1340 cm^−1^) increased in intensity. This indicated that fat and aromatic groups were exposed during desugarization. Polysaccharide material in the plant could hinder the adsorption ability of aromatic groups, therefore, it could concluded that removal of carbohydrates led to easier access to aromatic groups, which improved the adsorption capacity.

#### 3.2.4. Analysis of Surface Area

**Figure 6:**
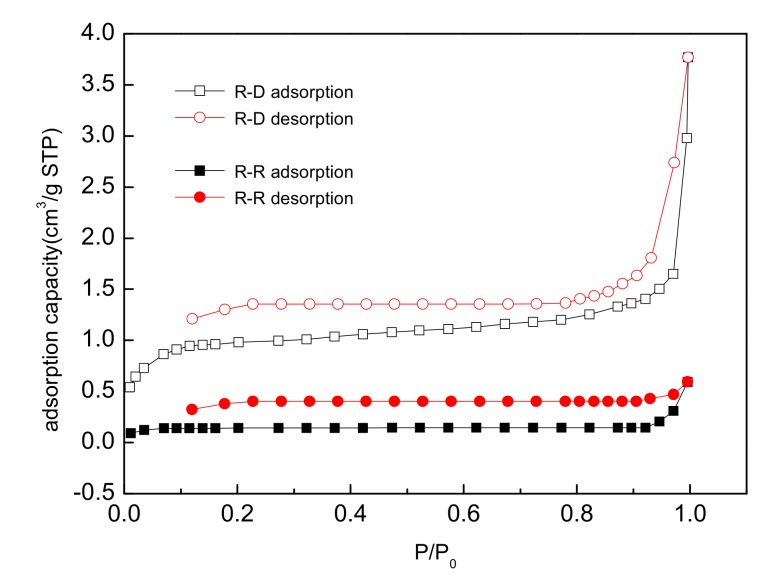
Adsorption-desorption isotherm.

Based on the experimental results, the adsorption capacity of roots was the largest reported above. Therefore, the surface area and pore analysis of the untreated and desugared samples of reed roots were studied. Adsorption-desorption N_2_ isotherms are shown in Figure 6, and the surface area and pore size data are listed in Table 7.

As shown in Figure 6, N_2_ adsorption desorption loops were not closed, because the experiments were below 0.3 Pa pressure, so irreversible adsorption occurred, and the adsorbed N_2_ could not be desorbed. This indicated that there was a strong adsorption potential in the micropores of the materials. The adsorption amount hardly increased with rising pressure, which indicated untreated roots could not absorb N_2_. While after desugarization, the adsorption amount increased which showed N_2_ was absorbed. 

As can be seen from Table 7, the surface area, pore area and micropore volume of desugared reed roots improved 14.27, 1.35 and 1.44 times respectively. This means that the adsorption capacity of desugared reed roots was significantly larger than that of untreated reed roots.

**Figure 7:**
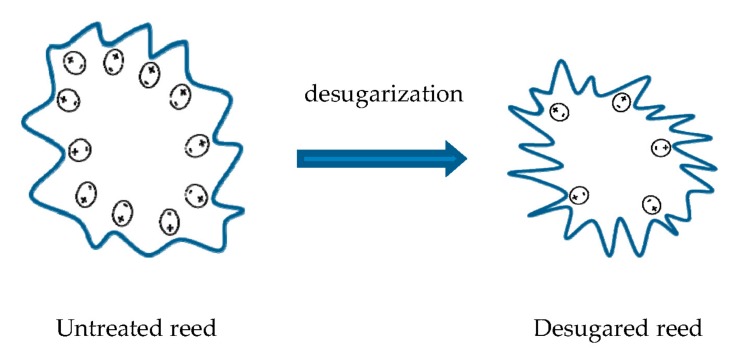
The adsorption mechanism schematic.

According to the analysis of the elemental composition, FTIR and surface area, the adsorption mechanism schematic is illustrated in Figure 7. The surface of untreated reeds was relatively smooth, while desugared reeds had rough surfaces with deep holes, and the polarity and hydrophilicity of untreated reeds were larger than that of desugared reeds. Thus the adsorption performance was stronger after desugarization.

## 4. Conclusions

The adsorption behavior and mechanism of fluoride ions by untreated and desugared reeds were studied. The following conclusions were drawn:

(1) Fluoride ions could be adsorbed by untreated reed residues, and the adsorption capacities were 191 mg/kg for roots, 175 mg/kg for stems and 150 mg/kg for leaves;
(2) Adsorption capacities after desugarization increased significantly by 8~12 times (roots 2136 mg/kg, stems 1551 mg/kg and leaves 1825 mg/kg);
(3) Desugared reed roots had high adsorption capacity for N_2_ compared with untreated roots that didn’t adsorb N_2_, and its surface area increased by 14.27 times. After desugarization, the surface area and aromaticity of reed residues increased, while the polarity and hydrophilicity decreased. This was the reason for the increase in the adsorption capacities.

The fluoride adsorption capacities of reed residues could be increased significantly after desugarization to levels competing with synthetic and metal-modified materials. In order to use this plant material in practical engineering, we will further explore the adsorption properties of fluoride ion in the presence of other anions such as sulphate, chloride, carbonate and bicarbonates that are commonly found.

## Figures and Tables

**Table 1:** Kinetic parameters.

Samples	Qe, Exp. (mg/kg)	Pseudo-First-Order Model			Pseudo-Second-Order Model		
		R^2^	K_1_ (1/min)	Qe, cal (mg/kg)	R^2^	K_2_ (kg/mg·min)	Qe, cal (mg/kg)
R-U	191.28	0.9764	0.0254	196.02	0.9263	1.38 × 10^−4^	226.52
S-U	175.29	0.9401	0.0170	183.88	0.8983	7.54 × 10^−5^	227.51
L-U	149.52	0.9208	0.0242	149.81	0.9348	1.90 × 10^−4^	170.91
R-D	2135.66	0.8932	0.0491	2108.14	0.9537	3.33 × 10^−5^	2291.83
S-D	1551.42	0.8668	0.0578	1530.64	0.9310	5.67 × 10^−5^	1646.98
L-D	1824.57	0.8318	0.0258	1840.13	0.8377	1.75 × 10^−5^	2074.31

**Table 2:** The fitted Parameters of Langmuir model and Freundlich model.

Samples	Langmuir Model				Freundlich Model		
	Q_m_ (mg/kg)	K_L_ (L/mg)	R^2^	R_L_	K_F_ (mg/kg) (L/mg) 1/n	n	R^2^
R-U	3547.04	0.0208	0.9401	0.4375	72.37	1.07	0.9351
S-U	655.37	0.0778	0.8418	0.4190	53.10	1.34	0.8181
L-U	668.87	0.0691	0.8116	0.4215	47.50	1.27	0.7867
R-D	10,859.82	0.1018	0.9882	0.4123	1210.72	1.50	0.9749
S-D	6404.78	0.0886	0.9801	0.4159	622.29	1.44	0.9695
L-D	5497.05	0.1460	0.9917	0.4016	869.47	1.71	0.9737

**Table 3:** Comparative investigation of fluoride adsorption using different absorbents.

Adsorbent		Isotherm Model	pH	Capacity (mg/g)	Reference
Natural materials	Natural pumice	F	6.0	4.50	[29]
	Natural pumice	F	3.0	1.170	[30]
	Natural geomaterial limonite (Iron Ore)	L	7.0	0.269	[31]
	Kaolinite clay	L		1.450	[32]
	Montmorillonites	F	6.0	3.365	[33]
	Untreated reed root	L	7.0	3.547	This study
	Untreated reed stem	L	7.0	0.655	This study
	Untreated reed leaf	L	7.0	0.669	This study
Modified materials	Modified pumice with FeCl_3_	F	3.0	21.740	[32]
	Modified pumice with HDTMA	F	3.0	25.000	[32]
	Modified magnetite ore with aluminum and lanthanum ions	L	7.8	M-Al 1.51 M-Na 1.42	[34]
	Modified montmorillonite with Fe(III)	L	4.5	9.696	[35]
	Modified chitosan with neodymium	L	7.0	22.380	[36]
	Modified zeolite with calcium chloride	F/L		1.766	[37]
	Desugared reed root	L	7.0	10.860	This study
	Desugared reed stem	L	7.0	6.405	This study
	Desugared reed leaf	L	7.0	5.497	This study
Synthetic materials	MnCO_3_ nanowires	L	7.0	11.580	[38]
	Graphene oxide (GO)-incorporated iron-aluminium mixed oxide	L	7.0	22.900	[39]
	Ce-Ti oxides nanoparticles	L	7.0	44.370	[40]
	Ce-Ti@Fe_3_O_4_ nanoparticles	L	7.0	91.070	[40]

**Table 4:** The fitted parameters of the Langmuir model at different temperatures.

Samples	Parameters	T (K)		
		298	308	318
R-U	Q_m_ (mg/kg)	3547.04	5313.88	5076.931
	K_L_ (L/mg)	0.0208	0.0169	0.0019
	R^2^	0.9401	0.9082	0.9879
S-U	Q_m_ (mg/kg)	655.37	648.05	1089.40
	K_L_ (L/mg)	0.0778	0.1271	0.0896
	R^2^	0.8418	0.7687	0.9161
L-U	Q_m_ (mg/kg)	668.87	604.28	947.65
	K_L_ (L/mg)	0.0691	0.1245	0.0933
	R^2^	0.8116	0.7465	0.8317
R-D	Q_m_ (mg/kg)	10,859.82	9573.71	11,775.19
	K_L_ (L/mg)	0.1018	0.1976	0.1835
	R^2^	0.9882	0.9796	0.9899
S-D	Q_m_ (mg/kg)	6404.78	10,470.99	10,280.32
	K_L_ (L/mg)	0.0886	0.0702	0.0957
	R^2^	0.9801	0.9832	0.9921
L-D	Q_m_ (mg/kg)	5497.05	6373.20	8185.86
	K_L_ (L/mg)	0.1460	0.1879	0.1771
	R^2^	0.9917	0.8436	0.9506

**Table 5:** The thermodynamic parameters.

Samples	T (K)	K_0_	ΔG (K·mol^−^^1^)	ΔH (KJ·mol^−1^)	ΔS (KJ·mol^−1^·K^−1^)
R-U	298	3.9785	−3.4213	4.6010	0.0270
	308	4.3751	−3.7794		
	318	4.4680	−3.9577		
S-U	298	3.4964	−3.1013	8.7097	0.0397
	308	3.9921	−3.5450		
	318	4.3598	−3.8929		
L-U	298	3.2021	−2.8834	8.3406	0.0378
	308	3.7504	−3.3849		
	318	3.9530	−3.6339		
R-D	298	6.9271	−4.7952	3.4746	0.0278
	308	7.4067	−5.1275		
	318	7.5623	−5.3490		
S-D	298	6.2228	−4.5295	3.6013	0.0273
	308	6.4882	−4.7885		
	318	6.8193	−5.0756		
L-D	298	6.5464	−4.6551	3.5335	0.0275
	308	6.9914	−4.9798		
	318	7.1577	−5.2036		

**Table 6:** Element composition and atomic ratio.

Samples	Qe, Exp. (mg/kg)	C (%)	H (%)	O (%)	H/C	(N + O)/C	O/C	K_d_ (L/kg)	Koc
R-U	191.24	42.46	6.06	44.59	1.71	0.81	0.79	63.67	149.95
R-D	2135.66	51.49	5.46	38.28	1.27	0.57	0.56	472.43	917.52
S-U	175.29	44.93	6.12	44.88	1.63	0.76	0.75	27.15	60.43
S-D	1551.42	50.96	5.91	43.43	1.39	0.64	0.64	262.42	514.95
L-U	149.52	42.13	6.10	40.43	1.74	0.76	0.72	26.52	62.95
L-D	1824.57	50.15	6.26	39.18	1.50	0.60	0.59	258.84	516.13

**Table 7:** The surface area, pore area, micropore volume and pore size of the untreated and desugared roots.

Sample Types	Surface Area (m^2^/g)	Pore Area (m^2^/g)	Micropore Volume (cm^3^/g)	Average Pore Size (nm)
R-U	0.1844	2.2274	0.0009	-
R-D	2.6321	3.0050	0.0013	8.8610
